# Call for a Dental Convention

**Published:** 1855-07

**Authors:** Elisha Townsend

**Affiliations:** President


### CALL FOR A DENTAL CONVENTION.

The undersigned, under the conviction that the profession of dentistry in the United States demands an organization of its cultivators and practitioners, adjusted in system, measures and spirit to the present state of the science and practice, respectfully invite the dentists throughout the Union, who concur with them in this belief, to meet in Convention, at the City of Philadelphia, on Thursday, the 2d day of August, 1855, at ten o'clock, A. M., for mutual consultation and deliberation upon the measures that may best promote harmony and efficiency of effort in the advancement of the common interest.

The changes which time has effected in the state of the profession since the existing associations were organized; the elevated standing which it has taken among the sister branches of remedial art; the vast improvement in its educational methods; the great numerical increase of practitioners, and, especially, the enlarged liberality of professional sentiment which has grown with its growth, all, as with one voice, call upon us to institute such a system of fraternal relations, and provide such instrumentalities as shall meet the wants of our condition.

No subsisting organization embraces a tythe of the great body of American dentists. Our proposition is, therefore, made to our brethren every where, to meet in the spirit of professional brotherhood, and organize such an association as shall be best fitted to satisfy the requirements of the fraternity, and give the best direction to its efforts for advancement in all usefulness and honor.

The Local Committee of Arrangement, whose names are subjoined, will charge themselves with the duty of providing the necessary accommodations for the Convention.

The invitation is to the dentists of the whole country, and they are requested, whether specially addressed, or by accident overlooked, to favor the Committee with their several apprehensions of the movement, and their wishes and purposes in reference to it.

Elisha Townsend, J. Gilliams, David W. Hogue, D. B. Whipple, H. S. Burr, J. DeHaven White, A. H. Briscoe, James S. Gilliams, James H. Briscoe, S. Dilingham, Joseph M'Ilhenney & Son, C. Newlin Pierce, John H. Githens, William H. Clark, S. L. Mintzer, Charles Townsend, jr., Daniel Neall, R. Arthur, James M. Harris, Henry Garrett, Charles A. DuBouchet, J. F. B. Flagg, Ely Parry, T. L. Buckinghan, A. R. Johnson, Edward Townsend, William W. Fouche, Henry Townsend, Jesse C. Green, J. R. M'Curdy, J. H. M'Quillen, C. B. Foster, John Waylan, U. B. Kirk, S. S. White, David Roberts, H. F. Reinstein, F. Coar, Henry Avery, Jethro J. Griffith, Samuel Marshall, John White, Louis Jack, C. S. Beck, Charles M. Slocum, S. R. Thatcher, Wm. Gorges, John M'Calla, James Fleming, J. W. Van Osten, T. W.

Walker, F. M. Dixon, Henry S. Porter, E. W. Haines, C. Moore, Samuel Welchens, Spencer Roberts, A. Homer Trego, William H. Thompson.

*Local Committee of Arrangement.*—Elisha Townsend, Robert Arthur, W. W. Fouche, H. S. Burr.

---

In pursuance of a resolution, passed by the American Society of Dental Surgeons, a special meeting will be held in Philadelphia, at the Dental College, on Wednesday the first day of August at 10 o'clock to consider the propriety of dissolving the Society.

ELISHA TOWNSEND, *President.*

May 18, 1855.

